# Molecular detection and genotyping of intestinal protozoa from different biogeographical regions of Colombia

**DOI:** 10.7717/peerj.8554

**Published:** 2020-03-09

**Authors:** Adriana Higuera, Ximena Villamizar, Giovanny Herrera, Julio Cesar Giraldo, Luis Reinel Vasquez-A, Plutarco Urbano, Oswaldo Villalobos, Catalina Tovar, Juan David Ramírez

**Affiliations:** 1 Grupo de Investigaciones Microbiológicas-UR (GIMUR), Departamento de Biología, Facultad de Ciencias Naturales, Universidad del Rosario, Bogota, Colombia; 2 Programa de Biología, Universidad INCCA de Colombia, Bogota, Colombia; 3 Centro de Estudios en Microbiología y Parasitología (CEMPA), Departamento de Medicina Interna, Facultad de Ciencias de la Salud, Universidad del Cauca, Popayan, Colombia; 4 Grupo de Investigaciones Biológicas de la Orinoquia, Unitrópico, Yopal, Colombia; 5 Hospital Local Santa María de Mompox, Programas Especiales (Lepra y TB), Mompox, Bolivar, Colombia; 6 Grupo de Enfermedades Tropicales y Resistencia Bacteriana, Universidad del Sinú, Monteria, Colombia

**Keywords:** *Giardia intestinalis*, *Cryptosporidium*, *Blastocystis*, *Entamoeba*, Molecular genotyping

## Abstract

**Background:**

Intestinal parasitic protozoa represent a serious problem of public health particularly in developing countries. Protozoa such as *Blastocystis, Giardia intestinalis*, *Entamoeba histolytica* and *Cryptosporidium* spp. are associated with diarrheal symptoms. In Colombia, there is little region-specific data on the frequency and circulating genotypes/species of these microorganisms. Therefore, the main objective of our study was to employ molecular detection and genotyping of *G. intestinalis* and *Blastocystis*, *Cryptosporidium* and *Entamoeba* spp. in samples from different biogeographical regions of Colombia.

**Methods:**

We collected 649 human fecal samples from five biogeographical regions of Colombia: the Amazon, Andean, Caribbean, Orinoco and Pacific regions. *Blastocystis, G. intestinalis, Cryptosporidium* spp. and *Entamoeba* complex were detected by microscopy and conventional PCR. Molecular genotyping was conducted to identify *Blastocystis* subtypes (STs) (18s), *G. intestinalis* assemblages (triose phosphate isomerase and glutamate dehydrogenase) and *Cryptosporidium* species (18s). Genetic diversity indices were determined using dnasp.5.

**Results:**

We detected *G. intestinalis* in 45.4% (*n* = 280) of samples, *Blastocystis* in 54.5% (*n* = 336) of samples, *Cryptosporidium* spp. in 7.3% (*n* = 45) of samples, *Entamoeba dispar* in 1.5% (*n* = 9) of samples, and *Entamoeba moshkovskii* in 0.32% (*n* = 2) of samples. *Blastocystis* STs 1–4, 8 and 9 and *G. intestinalis* assemblages AII, BIII, BIV, D and G were identified. The following *Cryptosporidium* species were identified: *C. hominis*, *C. parvum*, *C. bovis*, *C. andersoni*, *C. muris*, *C. ubiquitum* and *C. felis*. The Caribbean region had the highest frequency for each of the microorganisms evaluated (91.9% for *G. duodenalis*, 97.3% for *Blastocystis*, 10.8% for *Cryptosporidium* spp., 13.5% for *E. dispar* and 2.7% for *E. moshkovskii*). The Orinoco region had a high frequency of *Blastocystis* (97.2%) and the Andean region had a high frequency of *G. intestinalis* (69.4%). High and active transmission was apparent in several regions of the country, implying that mechanisms for prevention and control of intestinal parasitosis in different parts of the country must be improved.

## Introduction

Infectious diseases are major public health challenges worldwide. Despite efforts to reduce human morbidity and mortality, shortcomings in prevention and control measures continue to impact the continued transmission of pathogens in the human population, preventing the management of some diseases in endemic areas (Morens, Folkers & Fauci, 2004). Infectious diseases caused by intestinal parasites have a wide distribution worldwide. In 2001, approximately 3,500 million people were infected by protozoa and intestinal helminths where the children were the most affected (MinSalud, 2015) by protozoal infections. Members of the genus *Blastocystis* are the most common eukaryotic microorganisms in the human and animal intestine (Stensvold, Alfellani & Clark, 2012), followed by *Giardia intestinalis* (synonyms: *G. duodenalis* and *G. lamblia*) and various *Cryptosporidium* and *Entamoeba* species (Haque, 2007). Together, these are the main protozoan causative agents of diarrheal disease in humans worldwide (Caccio & Ryan, 2008; Haque, 2007; Jacobsen et al., 2007).

Worldwide, approximately 200 million individuals are infected by *Giardia* species, while the frequency of *Cryptosporidium* infection ranges from 0.1% to 10% in developed and developing countries, respectively (WHO, 2010). The frequency of amebiasis, caused mainly by *Entamoeba histolytica*, is often reported as near 20%, but can vary greatly depending on the region and the techniques used to differentiate the *E. histolytica*/*dispar*/*moshkovskii* complex (Silva et al., 2014; Tasawar, Kausar & Lashari, 2010). The frequency of *Blastocystis* ranges between 0.5% and 24% in industrialized countries and between 30% and 76% in developing countries (Wawrzyniak et al., 2013). However, other studies have identified populations of children where the frequency of *Blastocystis* approaches 100% (El Safadi et al., 2014). In Colombia, the latest national survey by the Ministry of Health revealed that *Blastocystis* was the most commonly identified protozoa in human feces, with a nationwide frequency of 52%. *Blastocystis* were followed by *Entamoeba* (17%), *Giardia* (15%) and *Cryptosporidium* (0.5%) spp. (MinSalud, 2015).

Molecular tools have been developed to assess the genetic diversity of protozoan parasites at the intra-species level. In the case of *G. intestinalis*, eight genotypes or assemblages (A–H) have been identified and are distributed worldwide. Within these assemblages, sub-assemblages (AI–AIII and BIII–BIV) have been established (Faria et al., 2017; Lasek-Nesselquist, Mark Welch & Sogin, 2010; Ryan & Cacciò, 2013). In Latin America, similar frequencies of assemblages A and B were observed in Brazil (Coronato Nunes et al., 2016) and Cuba (Pelayo et al., 2008), while the frequencies of the AI, AII, AIII, BIII and BIV sub-assemblages varied in Brazil, Argentina, Peru, Colombia and Mexico (Coronato Nunes et al., 2016; Minvielle et al., 2008; Molina et al., 2011; Perez Cordon et al., 2008; Sánchez et al., 2017; Torres-Romero et al., 2014). On the other hand, members of the genus *Blastocystis* can be classified into 17 subtypes (STs) (Stensvold et al., 2007) based on polymorphisms of 18S rDNA (Scicluna, Tawari & Clark, 2006). In humans, STs 1–3 are common in both Europe and South America (Del Coco et al., 2017; Malheiros et al., 2011; Ramírez et al., 2016; Santin et al., 2011), while ST4 is commonly found in Europe (Stensvold et al., 2011; Wawrzyniak et al., 2013) and was possibly associated with an enzootic cycle in nonhuman primates in Latin America (Ramírez et al., 2014; Santin et al., 2011). Approximately 20 different species have been identified within the genus *Cryptosporidium*, where *Cryptosporidium hominis* and *Cryptosporidium parvum* are the most common pathogens infecting humans (Feng & Xiao, 2017). Two markers (the small subunit of the ssuRNA and gp60) have been used to discriminate species and STs (Khan, Shaik & Grigg, 2018). Ten STs of *C. hominis* (Ia–Ik) and 16 STs of *C. parvum* (IIA–IIp) have been described (Garcia-R et al., 2017; Xiao, 2010). In Colombia, infections by *Cryptosporidium viatorum* (Sánchez et al., 2017) *Cryptosporidium galli* and *Cryptosporidium molnari* have been reported (Sánchez et al., 2018). Lastly, within the genus *Entamoeba*, the only pathogenic species is *E. histolytica*. However, the morphological similarities between the three species of the *E. histolytica*/*moshkovskii*/*dispar* complex (Pritt & Clark, 2008) make molecular tools required for species identification (Ximénez et al., 2009). A study in Colombia, in children under 16 years old, found a frequency of infections of 49.1%, being *E. dispar* the most frequently detected and *E. moshkovskii* also reported (López et al., 2015).

Colombia has a wide variety of climates and biogeographical regions classified according to epidemiological features. For instance, the biogeographical regions of Colombia are characterized by different climatic and ecosystem conditions, ranging from temperate zones to permanent snow in the mountain peaks. Moreover, the number of inhabitants and economic activities are increasing and the availability of resources are decreasing in some of these regions (IDEAM et al., 2007; Barón, 2002) affecting the ecological niches where some pathogens could be circulating. Also, variation in socioeconomic conditions may be associated with behavioral factors that drive the transmission of microorganisms through contact with animals from both urban and rural areas, as well as the consumption of food and water under inadequate sanitary conditions. All these features make Colombia a country where the transmission of intestinal microorganisms is very likely (MinSalud, 2015). For this reason, it is mandatory to establish intervention programs to know what protozoa are being transmitted, including their biological and molecular characteristics to improve control and prevention plans. Therefore, the main objective of this study was to conduct molecular detection and genotyping of *Giardia, Blastocystis, Cryptosporidium* and *Entamoeba* species from samples collected in different biogeographical regions of Colombia. We also compared the concordance results by PCR and microscopy in the analyzed samples.

## Methods

### Ethics approval and consent to participate

This study was a minimum risk investigation for participants. Both the ethical standards of the Colombian Ministry of Health (Youth Code) and the Helsinki Declaration of 2013 were followed. The parents or guardians of minors participating in the study signed informed consent forms and gave their permission to obtain samples. This study was approved by the research ethics committee of the Universidad del Rosario (registered in Act No. 394 of the CEI-UR), the ethics committee of the Department of Internal Medicine of the Universidad del Cauca (number VRI024/2016), and the INCCA University of Colombia (number 237894).

### Study area

Colombia is a country with significant geographical, ethnic, cultural and socioeconomic diversity. Based on climatic, territorial and ecosystem diversity, the country is subdivided into six natural regions: Insular, Caribbean, Amazon, Andean, Orinoco and Pacific. These regions are not precisely geographical but coincide with clusters that are the recipients of government budget funds. Except for the Insular region, all regions were included in this study.

The Caribbean region is located in the north of the country and includes seven departments. In the department of Córdoba, eight samples were obtained from Montería city and in the department of Bolívar, 30 samples were collected in the city of Mompós. This coastal region has some mountainous areas, contains both tropical and dry forest ecosystems, and is strongly influenced by the presence of bodies of water. The Amazon region has the smallest human population but the greatest diversity of flora and fauna. Its biome mainly comprises tropical forest and is characterized by warm weather and abundant precipitation. The human population is primarily indigenous. The Amazon region is located in southern Colombia and consists of six departments (Rangel-Ch & Aguilar, 1995). Fifty samples each were obtained from the departments of Guainía and Amazonas. The participating municipalities were Caño Conejo, Coco Nuevo, Coco Viejo and Puerto Inírida in Guainía and the cities of Leticia and Puerto Nariño in Amazonas.

The Andean region is the most populous in the country, housing half of the Colombian population. This region comprises the northern zone of the Andes and contains three mountain ranges that contribute to high climatic variability because of the different altitudes found within the region. The Andean region contains the departments of Antioquia, Boyacá and Cundinamarca; 50 samples were collected from each of these departments. The municipalities or cities contributing samples were Medellín, Río Negro, Amalfi, Bello, Caldas and El Santuario (Antioquia); Paipa, Villa de Leyva, Arcabuco, Tuta and Tunja (Boyacá) and the municipalities of Chaguaní, San Francisco, Fómeque, Soacha and the city of Bogotá (Cundinamarca). Other departments, including Quindío, Risaralda, Caldas and part of Tolima, make up the Coffee Axis. Another 50 stool samples were obtained from inhabitants of the municipalities of Calarcá, Armenia, Pereira, Córdoba and the Corregimiento Barcelona, all located within the Coffee Axis.

The Orinoco contains a large number of rivers, warm ecosystems and tropical and subtropical forests. This region is sparsely populated and comprises four departments. One of these is Casanare, where we collected 53 samples from the municipalities of Poré, Yopal and Tamara.

Finally, the Pacific region, one of the wettest in the world, is characterized by tropical forest and high species diversity. Although it is naturally resource rich, the region has poor urban development and infrastructure. The Pacific region contains four departments, one of these is the Cauca department, where 258 samples were collected from inhabitants of commune 8 in the city of Popayán. These samples were collected within the study of Villamizar and colleagues (Villamizar et al., 2019), and are, in turn, part of the current study.

### Study population

**Figure 1:**
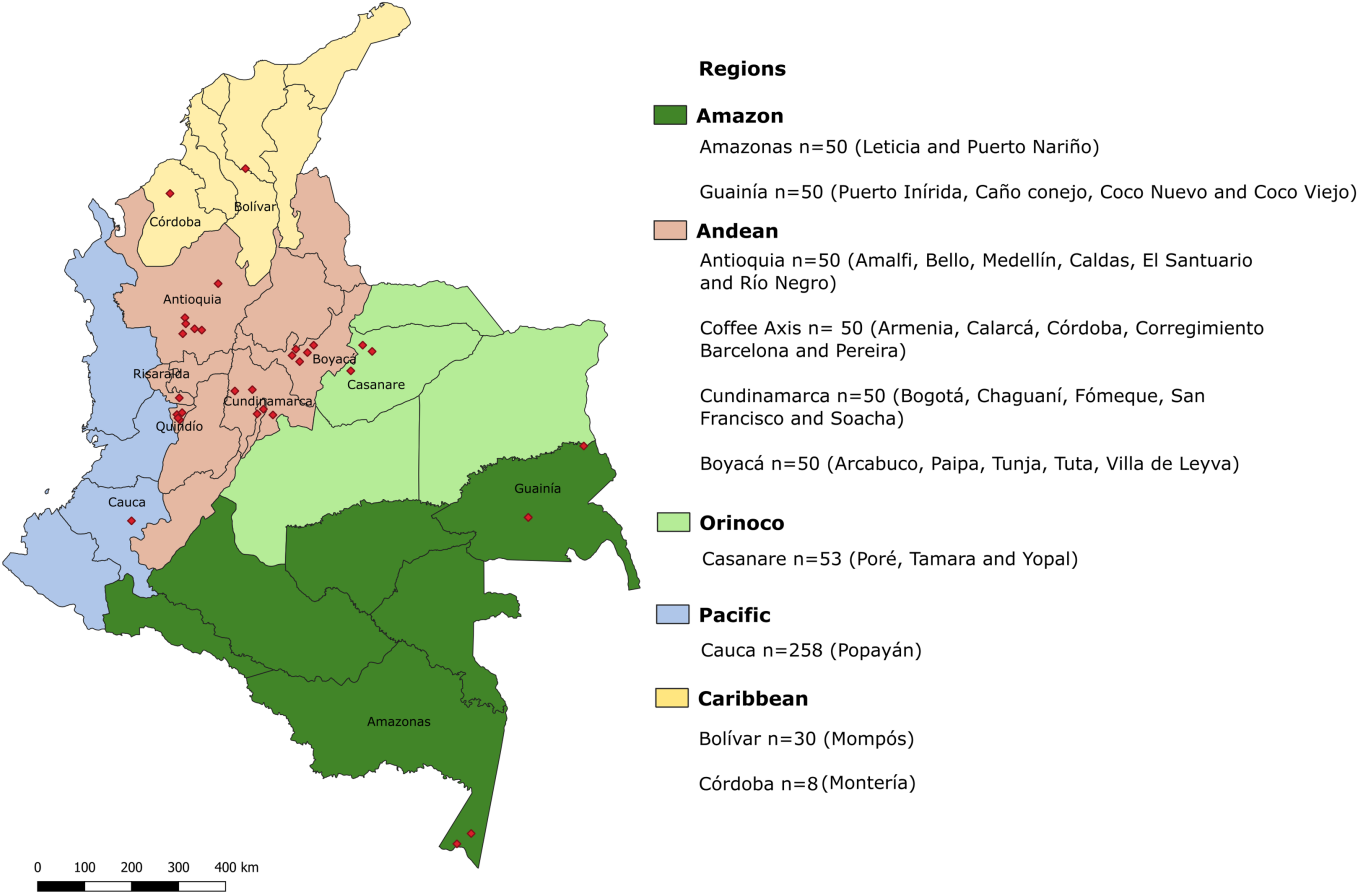
Geographic locations of regions in which samples were collected. Biogeographical regions of Colombia are indicated in colors. Each region is divided into departments. Red diamonds indicate the exact locations of sampling areas. In the legend, the departments sampled in each biogeographical region are indicated, along with the total number of samples for each department and the cities or municipalities from which the samples were obtained.

In total, 649 stool samples were collected from adults and children in different biogeographical regions of Colombia (Fig. 1). Convenience sampling was conducted to obtain samples from five different regions. The average age was 5 years (standard deviation: 6 years; range: 1–70 years). Microscopy is the gold standard used in Colombia to detect intestinal parasites, then most of the samples were evaluated by this diagnostic scheme following the protocol by Villamizar and collaborators (Villamizar et al., 2019), except for samples from Casanare, Bolívar and Córdoba, which could be only tested by PCR, since the entire sample portion was preserved in ethanol 100%. The individuals included in the study lived in both rural and urban areas of different municipalities/cities. The percentages of samples obtained in each biogeographical region were: Amazon (15.4%, *n* = 100), Andean (30.8%, *n* = 200), Caribbean (5.9%, *n* = 38), Orinoquía (8.2%, *n* = 53) and Pacific (39.8%, *n* = 258). The majority (86%, *n* = 558) of samples were assessed for the presence of intestinal protozoa by microscopy. All samples were subjected to molecular detection of intestinal protozoa and in those that were positive, further molecular characterization was conducted.

### DNA extraction

Prior to DNA extraction, approximately 300 μL of each sample was washed with sterile phosphate-buffered saline. Genomic DNA was extracted from stool samples using the Norgen Stool DNA Isolation Kit, Norgen Biotek Corp., following the recommendations of the manufacturer. During the lysis step, 10 μL of a recombinant plasmid, pZErO-2, was added (final concentration: 100 pg/μL). This plasmid contained the *Arabidopsis thaliana* aquaporin gene as an internal control for heterologous extrinsic amplification (Duffy et al., 2013).

### Conventional PCR

Initially, an internal amplification control (IAC) PCR was performed to verify that there was no inhibition of this technique using stool samples as template. All samples were subjected to this amplification control, except those from Cauca (The samples were collected and extracted directly in Popayan and therefore IAC was not added to the sample). Overall, 358 (91.6%) samples were validated with a positive amplification for the IAC, and were subjected to PCR to detect *G. intestinalis*, *Blastocystis*, *Cryptosporidium* and *Entamoeba* complex DNA. On the other hand, 33 (8.4%) samples that were negative for the IAC were discarded. IAC PCRs and molecular detection for *Giardia*, *Blastocystis* and *Cryptosporidium* spp., were performed in a final volume of 9 µL containing 3.5 µL of GoTaq Green Master Mix (Promega), 2 µL of template DNA, and primers. For IAC PCRs, primers were used at a final concentration of 0.4 µM. Species-specific primers were used at a final concentration of 1 µM. The sequences of the primers used for IAC were: IACFw (5′-ACCGTCATGGAACAGCACGTA-3′) and IACRv (5′-CTCCCGCAACAAACCCTATAAAT-3′) (Duffy et al., 2013). For species-specific PCR, the primers used were: *G. intestinalis*, forward (5′-CATGCATGCCCGCTCA-3′) and reverse (5′-AGCGGTGTCCGGCTAGC-3′) (Mejia et al., 2013); *Blastocystis*, FWD F5 (5′-GGTCCGGTGAACACTTTGGATTT-3′) and R F2 (5′-CCTACGGAAACCTTGTTACGACTTCA-3′) (Stensvold et al., 2012); and *Cryptosporidium*, CcF18s (5′-GTTTTCATTAATCAAGAACGAAAGTTAGG-3′) and CcR18s (5′-GAGTAAGGAACAACCTCCAATCTCTAG-3′) (Burnet et al., 2013). The thermal cycling parameters were as follows: 95 °C for 5 min; 40 cycles of 95 °C for 15 s, 58 °C for 1 min and 72 °C for 30 s; 72 °C for 10 min. Each PCR was performed individually.

For the *Entamoeba* complex, a conventional multiplex PCR was performed using previously reported conditions and primers EntF (5′-ATGCACGAGAGCGAAAGCAT-3′), EhR (5′-GATCTAGAAACAATGCTTCTCT-3′), EdR (5′-CACCACTTACTATCCCTACC-3′) and EmR (5′-TGACCGGAGCCAGAGACAT-3′) (Mahmoudi, Nazemalhosseini-Mojarad & Karanis, 2015). Differentiation between *E. histolytica, E. dispar* and *E. moshkovskii* was based on the size of the amplicon using these primers. DNA extracted from axenic cultures of each protozoan provided by The University of Texas Medical Branch were used as positive controls.

### Genotyping of *Giardia, Blastocystis* and *Cryptosporidium* spp.

Samples showing positive PCR amplification for *Giardia*, *Blastocystis* and *Cryptosporidium* spp. were subjected to conventional PCR to determine the assemblages, STs and species for each protozoan. For *Giardia*, two loci were amplified: glutamate dehydrogenase (*gdh*) and triose phosphate isomerase (*tpi*). To amplify *gdh*, primers GDHeF (5′-TCAACGTYAAYCGYGGYTTCCGT-3′), GDHiF (5′-CAGTACAACTCYGCTCTCGG-3′) and GDHiR (5′-GTTRTCCTTGCACATCTCC-3′) were used (Read, Monis & Thompson, 2004). To amplify *tpi*, primers Al3543 (5′-AAATIATGCCTGCTCGTCG-3′), Al3546 (5′-CAAACCTTITCCGCAAACC-3′), Al3544 (5′-CCCTTCATCGGIGGTAAATT-3′) and Al3545 (5′-GTGGCCACCACICCC-3′) were used (Sulaiman et al., 2003). *Blastocystis* STs and alleles were determined by amplifying a region of ssuRNAr using primers BhRDr (5′-GAGCTTTTTAACTGCAACAACG-3′) and RD5 (5′-ATCTGGTTGATCCTGCCAGT-3′) as reported previously (Scicluna, Tawari & Clark, 2006). To identify *Cryptosporidium* spp., a rRNA region was amplified using primers SSUrRNAF (5′-AGTGACAAGAAATAACAATACAGG-3′) and SSUrRNAR (5′-CCTGCTTTAAGCACTCTAATTTTC-3′) as described previously (Hunter et al., 2007).

Once all PCRs were performed, the size of each amplicon was assessed using 2% agarose gel electrophoresis followed by staining with SYBR Safe. Subsequently, each product was purified with ExoSAP-IT^®^ following the manufacturer’s recommendations. Both strands of each amplicon were sequenced using the Sanger method by Macrogen (Seoul, South Korea). Sequences were edited in MEGA 7.0 (Kumar, Stecher & Tamura, 2016) and compared with publicly available sequences using BLAST to verify that they corresponded to the expected taxonomic unit.

*Blastocystis* sequences were submitted to a database to identify STs and alleles (https://pubmlst.org/blastocystis/) (Jolley & Maiden, 2010). For *Giardia*, a multiple sequence alignment, including reference sequences for *gdh* and *tpi*, was performed using MUSCLE (Edgar, 2004) implemented in MEGA 7.0. A phylogenetic tree was constructed using maximum likelihood methods and 1,000 bootstrap replicates to determine assemblages. The accession numbers of the GenBank reference sequences used for *gdh* were: AI (M84604.1), AII (AY178737.1), AIII (EU637582.1), BIII (AF069059.1), BIV (AY178739.1), C (U60982.2), D (U60986.2), E (AY178741.1), F (AB569384.1), G (AF069058.2) and H (GU176089.1). Reference sequences contained in GenBank with the following accession numbers for *tpi* were used: AI (AF069556 .1), AII (AF069557.1), AIII (DQ650648.1), BIII (AF069561.1), BIV (AF069560.1), C (AF069563.1), D (DQ246216.1), E (AY228645. 1), F (AF069558.1) and G (EU781013.1). As outgroup, reference sequences of *G. ardeae* (AF069060.2) for *gdh* and *G. microti* (AY228649.1) for *tpi* were used. *Cryptosporidium* species were determined by comparing target sequences with sequences available in GenBank.

### Indices of genetic diversity

To assess the degree of DNA polymorphism, we constructed a multiple alignment of concatenated sequences for each of the loci evaluated for both *G. intestinalis* and *Blastocystis* using MAFFT v7. For the *gdh* and *tpi* loci of *G. intestinalis*, we analyzed 30 (295 sites including gaps) and 25 (465 sites including gaps) sequences, respectively. In case of *Blastocystis* with the 18s gene, we analyzed 114 (1,635 sites including gaps) sequences. All these sequences were used to calculate the indices of diversity (π and Θ), number of polymorphic (segregating) sites (*S*), number of haplotypes (*h*), and the haplotype diversity by department. DnaSP v5 software was used for these analyses.

### Statistical analysis

Data were summarized using univariate statistics in Stata 14 (StataCorp, 2015, Stata Statistical Software: Release 14). Subsequently, Cohen’s kappa indices were calculated to assess agreement between the results of microscopy and molecular techniques, both globally and for each of the parasites individually.

## Results

### Sample description and detection of protozoa

The ages of individuals from which samples were collected ranged between 1 and 70 years (average, 4.8 years; standard deviation, 5.5 years). The largest number of samples (39.8%) were collected in the Pacific region (Department of Cauca), while 30.8% were collected in the Andean region (Departments of Antioquia, Boyacá, Cundinamarca and the Coffee Axis). The majority (74.9%) of samples came from rural areas.

### Comparison of protozoan detection by microscopy and PCR

The majority of samples were positive by microscopy (68.3%) and molecular methods (71.2%). The frequency of positive samples by PCR (*n* = 616) *vs* microscopy (*n* = 649) was calculated for each protozoan: *G. intestinalis* (PCR 41.1% *vs* microscopy 24.5%), *Blastocystis* (PCR 49.0% *vs* microscopy 33.6%), *Cryptosporidium* (PCR 5.6% *vs* microscopy 27.3%), and the *Entamoeba* complex (PCR 22.9% *vs* microscopy 0.2%). The concordance between direct microscopy and by conventional PCR was analyzed both globally and for each protozoan. In all cases, a low concordance between detection techniques was observed, with kappa indices of 0.3807 for detection of all protozoa and 0.2699, 0.1478, 0.0149 and −0.0036 for *G. intestinalis*, *Blastocystis*, *Cryptosporidium* spp. and the *Entamoeba* complex, respectively.

### Frequency of protozoa

In total, 558 samples were assessed by microscopy. Of these 558 samples, 25.4% were assessed for *G. intestinalis* (*n* = 142), 33.9% for *Blastocystis* (*n* = 189), 23.1% for members of the *Entamoeba* complex (*n* = 129), 27.2% for *Entamoeba coli* (*n* = 152), 15.9% for *Cryptosporidium* spp. (*n* = 89), 12.3% for *Cyclospora* spp. (*n* = 69), 5.7% for geohelminths such as *Strongyloides stercoralis* (*n* = 32), 5.0% for *Ascaris lumbricoides* (*n* = 28), 4.3% for *Trichuris trichiura* (*n* = 24) and 4.6% for *Uncinaria* (*n* = 26).

### Giardia intestinalis

**Figure 2:**
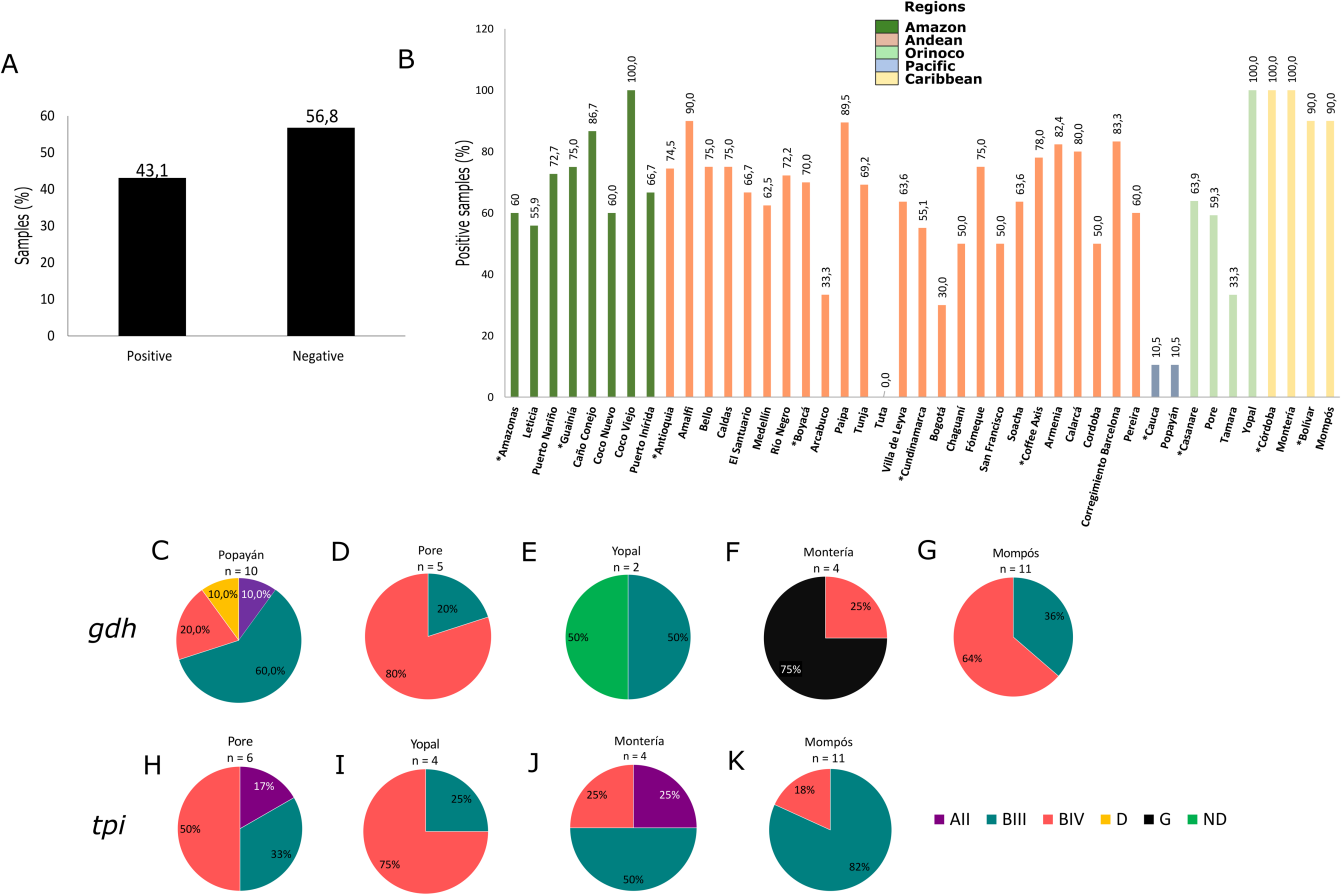
Frequency and assemblages for *G. intestinalis*. (A) Total percentage of positive and negative samples for *G. intestinalis*. (B) Frequency of positive samples for *G. intestinalis* by region. Departments indicated by * are highlighted. Colors indicate the biogeographical regions to which the sampled areas belong. (C–G) Frequencies of assemblages obtained in selected cities of some regions for the *gdh* locus. (H–K) Frequencies of assemblages obtained in selected cities of some regions for the *tpi* locus.

Using molecular detection by PCR, 43.1% of samples tested positive for *G. intestinalis* (Fig. 2A). The Caribbean region showed the highest frequency at 89.5% (95% CI [83.1–100.7]), followed by the Andean region (mainly the Department of Antioquia and the Coffee Axis) and the Amazon region (municipalities of Coco Viejo and Caño Conejo, Department of Guainía) in which the majority of sampled areas had a frequency greater than 60% (Table 1; Fig. 2B). Certain areas such as Yopal of Casanare and Paipa of Boyacá were distinguished by their extremely high *G. intestinalis* frequency (89.5% and 100%, respectively). From positive samples, assemblages were identified using the *gdh* marker for 33 samples as follows: AII (3.0%), BIII (36.3%), BIV (48.8%), D (3.0%) and G (9.1%). Sub-assemblage BIV was the most frequent, mainly in the municipality of Poré (Casanare), followed by sub-assemblage BIII with significant frequency in the cities of Yopal (Casanare) and Popayán (Cauca); the lattermost city had the greatest variety of assemblages. In the city of Monteria, a high frequency of assemblage G (75%) was observed. Twenty-five samples were genotyped using the *tpi* marker, and the AII, BIII and BIV assemblages were detected at frequencies of 8%, 56% and 36%, respectively. The highest frequency (82%) was observed for the BIII sub-assemblage in Mompós, followed by the BIV sub-assemblage (75%) in the city of Yopal (Figs. 2C–2K). From one sample collected in the city of Yopal, we found an inconsistency with the assigned assemblages using different markers. In the case of *gdh* marker, this sample clustered between AI and AII sub-assemblages, and could not be determined its assemblage with *gdh*, but with *tpi* marker this sample clustered with the BIV sub-assemblage.

**Table 1:** Prevalence of protozoa assessed by PCR in each biogeographical region.

Regions	*n*	*G. intestinalis*	*Blastocystis*	*Cryptosporidium* spp.	*E. dispar*	*E. moshkovskii*
Amazon	100	60.0%	49.0%	5.0%	0%	0%
		(*n* = 60)	(*n* = 49)	(*n* = 5)		
		95% CI [56.2–74.9]	95% CI [48.7–68.1]	95% CI [1.1–10.2]		
Andean	200	68.0%	58.0%	5.5%	0%	0%
		(*n* = 136)	(*n* = 116)	(*n* = 11)		
		95% CI [64.8–77.4]	95% CI [53.6–67.2]	95% CI [2.03–8.1]		
Caribbean	38	89.5%	94.7%	10.5%	13.1%	2.6%
		(*n* = 34)	(*n* = 36)	(*n* = 4)	(*n* = 5)	(*n* = 1)
		95% CI [83.1–100.69]	95% CI [92.1–102.5]	95% CI [0.8–20.8]	95% CI [2.5–24.5]	95% CI [−1.9 to 12.7]
Orinoco	53	43.4%	66.0%	0%	7.5%	0%
		(*n* = 23)	(*n* = 35)		(*n* = 4)	
		95% CI [29.9–56.1]	95% CI [61.5–70.5]		95% CI [−1.6 to 15.6]	
Pacific	258	10.5%	38.76%	9.70%	0%	0.39%
		(*n* = 27)	(*n* = 100)	(*n* = 25)		(*n* = 1)
		95% CI [6.8–14.3]	95% CI [33.2–45.2]	95% CI [6.2–13.4]		95% CI [−0.4 to 1.2]

**Note:**

*n*, number of samples; 95% CI, 95% confidence interval.

### Blastocystis

**Figure 3:**
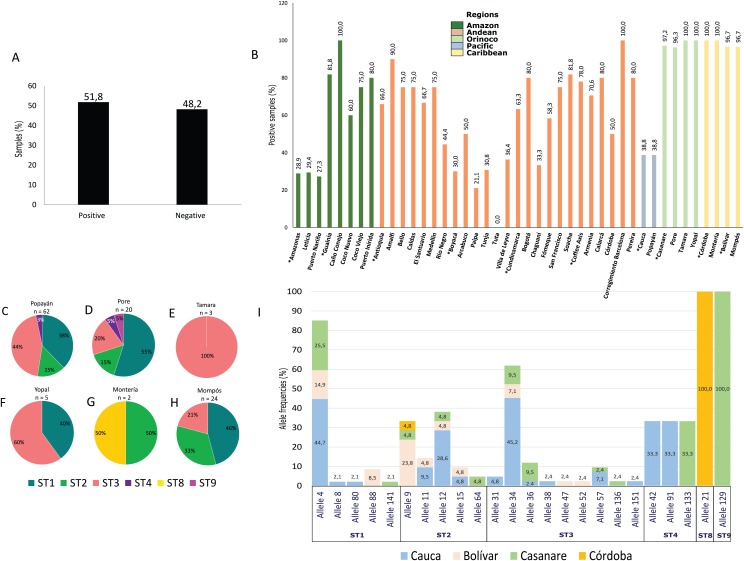
Frequency, STs and alleles for *Blastocystis*. (A) Total percentage of positive and negative samples for *Blastocystis*. (B) Frequency of positive samples for *Blastocystis* by region. Departments indicated by * are highlighted. Colors indicate the biogeographical regions to which the sampled areas belong. (C–H) Frequencies of STs obtained in six selected cities from Pacific, Orinoco and Caribbean regions for the 18s locus. The raw data is supplied. (I) Allele frequencies by ST and by department.

Of the samples analyzed, 51.8% were positive for *Blastocystis* by PCR (Fig. 3A). The Caribbean region showed the highest frequency of *Blastocystis* at 94.7% (95% CI [92.1–102.5]), followed by the Orinoco and the Andean regions (Table 1). In the Coffee Axis, the Corregimiento region of Barcelona, Amalfi in the Department of Antioquia and Caño Conejo in the department of Guainía, high frequency rates of 100%, 100% and 90% were observed, respectively (Fig. 3B). A total of 116 samples were subtyped from Popayán, Poré, Tamara, Yopal, Montería and Mompós. STs 1 (41.4%, *n* = 48), 2 (18.1%, *n* = 21), 3 (36.2%, *n* = 42), 4 (2.6%, *n* = 3), 8 (0.9%, *n* = 1) and 9 (0.9%, *n* = 1) were detected. The Poré (Casanare) and Popayán (Cauca) regions showed the greatest diversity of STs (Figs. 3C–3H). Greater allelic diversity was found within ST3 and the most frequently observed allele in Cauca was 34, followed by allele 57 and then other alleles including 31, 36, 38 and 151. For ST1, the most frequent allele was 4 in Cauca, Bolívar and Casanare, although alleles 8, 80, 88 and 141 were also observed. For ST2 the most frequent alleles were 9 in Bolívar and 12 in Cauca, although alleles 11, 15 and 64 were also detected. Noteworthy findings included the detection of alleles 42 and 91 in Cauca, ST4 allele 133 in Casanare, ST8 allele 21 in Córdoba and ST9 allele 129 in Casanare (Fig. 3I). The lattermost finding represents the first report of this allele in human samples from Colombia.

### Diversity indices

Genetic diversity indices by department were calculated based on these alignments. For *G. intestinalis*, the number of segregating (polymorphic) sites (*S*) was 202 for *gdh* and 105 for *tpi*, with haplotypic diversities of 0.977 and 0.903, respectively. The nucleotide diversity indices π and Θ, as well as haplotypic diversity (Hd), were high for the population in Córdoba for both loci and in Casanare for *tpi*. The lowest diversity in *gdh* was found among sequences from Cauca. Unfortunately, *tpi* sequences from Cauca showed electropherograms of poor quality and were not analyzed.

For *Blastocystis* sequences, the departments of Bolívar and Córdoba showed a greater number of polymorphic (segregating) sites (*S*). In particular, the Bolívar sequences showed the highest number of haplotypes (24), with a haplotypic diversity of 0.989 and higher nucleotide diversity indices compared with Casanare and Cauca. The latter had the lowest sequence diversity (Table 2).

**Table 2:** Genetic diversity indices of *G. intestinalis* and *Blastocystis* by department.

	Locus	Parameter	Bolívar	Casanare	Córdoba	Cauca	Total
*G. intestinalis*	gdh	*n*	11	7	4	8	30
		π	0.08719	0.16324	0.31284	0.05862	0.18028
		Θ	0.14248	0.20964	0.34426	0.0736	0.3224
		*S*	95	121	141	47	202
		*h*	11	7	4	4	24
		Hd	0.891	1	1	0.75	0.977
		SD	0.039	0.076	0.177	0.139	0.017
	tpi	*n*	11	10	4	ND	25
		π	0.00689	0.04833	0.11351	ND	0.03922
		Θ	0.01228	0.08307	0.12426	ND	0.07113
		*S*	15	95	93	ND	105
		*h*	5	9	4	ND	17
		Hd	0.618	0.978	1	ND	0.903
		SD	0.164	0.054	0.177	ND	0.054
*Blastocystis*	18s	*n*	24	28	2	60	114
		π	0.27703	0.14334	0.48703	0.08725	0.71859
		Θ	0.35406	0.27501	0.48703	0.21444	0.56508
		*S*	209	84	169	6	19
		*h*	24	22	2	5	90
		Hd	0.989	0.966	1	0.219	0.993
		SD	0.012	0.024	0.5	0.07	0.03

**Note:**

*n*, number of sequences; π, nucleotide diversity; Θ, theta (per site) from eta; *S*, number of segregating sites; *h*, number of haplotypes; Hd, haplotype diversity; SD, standard deviation; ND, not determined.

### *Cryptosporidium* and *Entamoeba* spp.

From the samples tested using PCR, 6.9% (*n* = 45) were positive for *Cryptosporidium* spp. Frequency was higher in the Caribbean (10.5%) and Pacific (9.7%) regions of Colombia (Table 1). We were able to identify species from 15 samples: 25% (*n* = 4) corresponded to *C. parvum*, 12.5% (*n* = 2) to *C. hominis*, 18.7% (*n* = 3) to *C. andersoni*, 25% (*n* = 4) to *C. muris* and 6.2% (*n* = 1) each to *C. ubiquitum* and *C. felis*. Samples where we identified *Cryptosporidium* spp. came from the cities of Popayán (*C. hominis* and *C. parvum*), Leticia (*C. andersoni*), Paipa (*C. muris* and *C. andersoni*) and the Departments of Antioquia (*C muris*, *C. andersoni* and *C. ubiquitum*), Bolívar (*C. hominis* and *C. muris*) and Córdoba (*C. felis*). As for members of the *Entamoeba* complex, 1.4% (*n* = 9) of samples tested positive for *E. dispar* in the departments of Bolívar (Mompós) and Casanare (Poré and Yopal) and 0.5% (*n* = 3) tested positive for *E. moshkovskii* in the departments of Cauca (Popayán) and Bolívar (Mompós). No samples were positive for *E. histolytica* (Table 1). All sequences were deposited on GenBank under the accession numbers MN877659–MN877714.

## Discussion

Colombia is a privileged country with natural wealth, geographical variety and ecosystem diversity. However, the climatic conditions and location of the country, in addition to the unequal distribution of resources in different regions, give rise to some primarily rural areas with unfavorable socioeconomic conditions and inadequate sanitary conditions. These factors directly influence the transmission of parasitic diseases among the residents of a given region (Ortiz, López & Rivas, 2012). Another factor that plays a major role in transmission of infectious protozoa is age: children tend to be the most common hosts and adults are likely to be an important source of transmission to children (Carvajal-Restrepo et al., 2019). Age may be associated with susceptibility to infection due to age-dependent immunological conditions that favor colonization by protozoa as well as age-dependent malnutrition and behavioral factors that affect transmission (Harhay, Horton & Olliaro, 2010). Likewise, these factors can influence the transmission of helminths, which explains the finding of some of them in the samples evaluated by microscopy. It is important to clarify that although the objective of our study was not the detection of these geohelminths, we wanted to report them due to the great importance they have mainly in the child population, associated not only with immunological and malnutrition problems but also with growth and development (Papier et al., 2014). Our findings also support a high transmission rate of helminths in the country, which has severe implications in control programs across the country.

Our results showed a high frequency of intestinal protozoa present in different regions of the country. Using microscopic detection (with the exception of the Caribbean and Orinoco), we observed that the Andean region had the highest frequency of *G. intestinalis*, *Blastocystis*, members of the *Entamoeba* complex and *Cryptosporidium* spp. Using molecular tests, the region with the highest frequency of all protozoa evaluated was the Caribbean. There was a low concordance (kappa index = 0.38) between the two techniques evaluated (microscopy and PCR). However, it is not possible to assert that the Andean or Caribbean regions truly had higher frequency of these protozoa, as our study had an important selection bias: sampling was carried out at convenience, with a higher number of samples obtained in regions such as the Andean and Pacific regions. However, it is important to note that the Andean region concentrates the largest population in Colombia and the Pacific region is one of the rainiest areas in the world, with low economic conditions, inadequate health conditions and poor access to education which might to some extant explain our findings (Barón, 2002). Thus, future studies would be necessary to collect a larger number of samples of comparable quantity in each region. Despite this, our results are in agreement with those of a survey conducted using microscopy by the Ministry of Health in 2015 (MinSalud, 2015). For example, using molecular tests, we observed that the areas with higher frequencies of these protozoa coincided with the Caribbean region and the Andean region in most cases, with the exception of *G. intestinalis*, because in the survey by the Ministry of Health, was observed at higher frequency in the Colombian Amazon.

In Colombia, most reports on protozoan pathogens have focused solely on microscopic detection (Agudelo-Lopez et al., 2008; Carvajal-Restrepo et al., 2019). Several studies have shown differences in detection rates using molecular tests, which allow identification of cryptic species and their genotypes in addition to detection (Morgan et al., 1998; Stensvold et al., 2018). Thus, there is clear value in using complementary techniques (Beyhan & Taş Cengiz, 2017; Sri-Hidajati et al., 2018; Mateo et al., 2014) for molecular epidemiological studies, which may help to better elucidate the transmission dynamics of microorganisms and to establish better prevention and control plans. Another advantage of using molecular techniques is their sensitivity in cases of polyparasitism (Meurs et al., 2017). Polyparasitism is an important factor in the transmission of parasitic diseases, and the presence of different infectious agents, including helminths and protozoa, may serve as an indicator of inadequate sanitary conditions, immune suppression, nutritional deficiencies and continual reinfection (Supali et al., 2010). In our study, 29.3% of samples evaluated were positive for both *Blastocystis* and *G. intestinalis*, 1.7% were positive for *Blastocystis*, *G. intestinalis* and *Cryptosporidium* spp., 3.4% were positive for *G. intestinalis* and *Cryptosporidium* spp., and 3.4% for *Blastocystis* and *Cryptosporidium* spp. The remaining co-infection combinations occurred at less than 1.4%. None of these combinations showed any geographical associations.

Few studies of these protozoan pathogens have been conducted in Colombia. Studies of samples from indigenous communities in the Amazon (Sánchez et al., 2017), from a rural region in La Vírgen (Ramírez et al., 2015), and from children in rural schools in the municipality of Apulo (Hernández et al., 2019), Cundinamarca, found *G. intestinalis* in human fecal samples. Sub-assemblages AI, AII, BIII and BIV and sub-assemblages AII, BIII and BIV were detected in the feces of children in nurseries of the Colombian Institute of Family Welfare. Assemblages C and D were detected in samples from dogs in Tolima (Rodríguez et al., 2014). These results are consistent with the detection of assemblages AII, BIII and BIV in the Orinoco, Pacific and Caribbean regions in our study, with the exception of the presence of assemblage D in the Pacific and assemblage G in the Caribbean (Figs. 2C–2K). As in other studies outside Colombia, we observed no restriction of assemblages to specific geographic regions (Broglia et al., 2013; Feng & Xiao, 2011). Assemblage D is typically associated with dogs, while assemblage G infects rodent including rats and mice (Caccio & Ryan, 2008). Thus, there is the potential for human infection by these assemblages in humans and they could potentially maintain an active cycle of transmission or generate transient infections, in humans (Heyworth, 2016). The association between these assemblages and the development of disease is not clear (Sprong, Cacciò & Van Der Giessen, 2009), but they may be acquired through the consumption of untreated water in rural regions where potable drinking water systems are absent, allowing closer contact with animal feces and increasing the risk of zoonotic transmission (Fantinatti et al., 2016). In agreement with this, assemblage H was detected in a study of water supplied by treatment plants in Nariño (southwest Colombia) (Sánchez et al., 2018), suggesting that water or the feces of wild animals that have not been studied as possible reservoirs could explain the presence of these assemblages. It is also important to consider that in the Caribbean region consumption of exotic animals and animal products, including iguana eggs, small crocodiles, freshwater turtles and armadillos, is very common. The potential role of these foods in the transmission of infections is unknown.

Another protozoan detected with high frequency was *Blastocystis*, mainly in the Caribbean and Orinoco regions. Frequency rates were often above 80%, especially in some regions of the Amazon such as Caño Conejo, Puerto Inírida (Guainía), of the Andean region such as Amalfi (Antioquia), the city of Bogotá, Soacha (Cundinamarca) and Calarcá, Corregimiento Barcelona, Pereira (Coffee Axis) (Fig. 3B). These findings are in agreement with results obtained using microscopy in Colombia that showed significant frequency in Caribbean regions such as Santa Marta (62.6%) and in Andean areas such as Santander (25%), Bogotá (22.4%), Quindío (36.4%) and Cundinamarca (34.8%) (Londono-Franco et al., 2014). When performing subtyping of *Blastocystis*, we identified STs 1–4, 8 and 9, with STs 1–3 having the highest frequency as reported by Del Coco and collaborators in a review made in 2017 and another study in Brazil (Del Coco et al., 2017; Malheiros et al., 2011). The municipality of Poré, Casanare showed the greatest diversity of STs. Similarly, the lower proportion of ST4 observed in the Caribbean and in the Pacific coincides with previous reports suggesting that this subtype is of recent origin in humans from the Americas (Stensvold et al., 2012) and of ethnic origin in Colombia associated with the enzootic cycle (Jiménez, Jaimes & Ramírez, 2019; Ramírez et al., 2014; Santin et al., 2011). Surprisingly, one sample was positive for ST8 in the Caribbean and another for ST9 in Casanare in the Orinoco region; these STs are rarely detected in humans (Stensvold & Clark, 2016). This is the first report of ST9 in Colombia, previously, one study in Italy reported the presence of ST9 in samples from symptomatic humans (Meloni et al., 2011). However, more studies are required to evaluate the potential zoonotic origin of this ST and its relationship with the presence of symptoms (Stensvold et al., 2009). A previous study reported the presence of ST8 in Colombia in marsupial stool samples (Ramírez et al., 2014), while another study detected this ST in arboreal nonhuman primates in Asia and South America (Alfellani et al., 2013). Few studies have reported the presence of this ST in humans, but it could apparently be involved in zoonotic transmission to humans (Meloni et al., 2011; Stensvold et al., 2007), where a great variety of animals could be involved in the transmission. This is because there is a great diversity of fauna and ecosystems in the country. For instance, in the Caribbean and Orinoco regions exist diverse ecosystems including savannah, mountainous forest, bodies of water, jungles and moorland (Barón, 2002; IDEAM et al., 2007; Vergara, 2018), these are exploited by each department to generate economic resources, and the presence there of nonhuman primates, rodents, birds and pigs infected with intestinal protozoa could increase the risk of zoonotic transmission in rural areas as has been reported in the country and in Brazil (Rondón et al., 2017; Valença-Barbosa et al., 2019).

We also characterized the alleles of each of the STs. No geographical associations were observed for STs or alleles. Allele 4 of ST1 was detected in the regions of Cauca (44.7%), Casanare (25.5%) and Bolívar (14.9%) (Fig. 3I), and was the most frequently observed as previously reported (Ramírez et al., 2014; Sánchez et al., 2017). In addition, alleles 8, 80, 88 and 141 were found in ST1, alleles 9, 11, 12, 15 and 64 within ST2, and alleles 31, 34, 36 were detected in ST3. The presence of alleles 38, 47, 52, 57, 136 and 151 provided evidence of the great intra-subtype diversity present and mostly agreed with studies of STs circulating in Ecuador, Peru, Bolivia, Colombia, Brazil and Argentina in samples from humans, domestic animals and the enzootic cycle (Ramirez et al., 2017, 2014, 2016). For ST4, alleles 42, 91 and 133 were identified, which had previously been reported in Colombia; in particular, allele 91 that is possibly of European origin (Ramírez et al., 2014; Stensvold et al., 2011). For ST8 isolates reported in Colombia and Brazil (Ramírez et al., 2016), the 21 allele probably had a zoonotic origin. For ST9 we detected allele 129, of which there is no previous report in Colombia. The origin of this ST has not been established, and then it is not possible to make inferences about its transmission. As mentioned above, great diversity was present among the STs characterized for *Blastocystis*, and establishing the transmission dynamics for several of the STs detected at low frequency would be a useful task.

In addition to detecting protozoa and determining the frequencies of STs and assemblages, the genetic diversity among sequences of *G. intestinalis* and *Blastocystis* was evaluated by department and by marker. For *G. intestinalis*, diversity indexes were higher for assemblages from the Casanare and Córdoba departments for *gdh* (Table 2). However, the low number of sequences obtained from the Córdoba region means that the degree of diversity would need to be verified using more samples in a future study. For *Blastocystis*, greater diversity was found in the Caribbean region in the departments of Bolívar and Córdoba. This was expected since *Blastocystis* usually has high inter-subtype variability (Stensvold et al., 2012), as reported in a study of SSU DNAr genes conducted in Mexico, where the results showed similar diversity indices within each subtype, despite their different geographical regions and different inter-subtype indices (Villegas-Gómez et al., 2016), and a greater diversity between the STs of a control group compared with one associated with irritable bowel syndrome (Vargas-Sanchez et al., 2015). Like *G. intestinalis*, the number of sequences for Córdoba was very small, avoiding any strong conclusions regarding this population.

Finally, in the case of other less frequently detected protozoa such as *Cryptosporidium* and *Entamoeba* spp., we identified in some cases the species present in positive samples. For *Cryptosporidium* spp., *C. andersoni* was detected in the Amazon; *C. muris, C. ubiquitum* and *C. andersoni* were detected in the Andean region; *C. hominis*, *C. muris* and *C. felis* were detected in the Caribbean; and *C. hominis* and *C. parvum* were detected in the Pacific. These results agreed with previous studies (Galván-Díaz, 2018; Sánchez et al., 2017, 2018), except for *C. ubiquitum* and *C. andersoni* which had not previously been reported in human feces in Colombia. These two species are associated with a wide variety of animal hosts including domestic and wild ruminants, rodents, omnivores and primates (Fayer, Santín & Macarisin, 2010). The low host specificity of *C. ubiquitum* along with the shared habitats of different animals can contribute to its wide distribution and therefore to possible infections in humans, especially immunocompromised patients (Fayer, Santín & Macarisin, 2010). *C. parvum* and *C. hominis* are the species most frequently detected in humans. However, cattle and other domestic and wild animals infected with different species can have great importance in public health and the transmission of this parasite (Ryan, Fayer & Xiao, 2014). The detection of species associated with bovine hosts and cats infecting humans allows us to infer that the transmission of *Cryptosporidium* spp. in the regions evaluated is zoonotic and possibly also from human to human. Likewise, these findings suggest the great need to evaluate prevention and control measures for parasitic infections and the need to improve water sanitation infrastructure for human consumption.

For the *Entamoeba* complex, we only obtained samples positive for *E. dispar* in the departments of Bolívar and Casanare and samples positive for *E. moshkovskii* in Bolívar. This indicated probable orofecal transmission in the areas evaluated. Our findings are consistent with a study conducted by Lopez et al. showing a high frequency of *E. dispar* and *E. moshkovskii* in La Virgen, Cundinamarca, Colombia and low frequency of *E. histolytica* (López et al., 2015). Due to the low number of samples detected for *Cryptosporidium* and *Entamoeba* spp., it was not possible to establish any type of geographical associations for these parasites. For these microorganisms, the number of samples collected in each region should be expanded to establish with greater certainty the true frequencies of the circulating variants in the country.

## Conclusions

In conclusion, ours is the first study to assess the frequency and genotypes of intestinal protozoa using sampling areas located in five biogeographical regions of Colombia. Our results showed frequent transmission of intestinal protozoa and high genetic diversity of *G. intestinalis* and *Blastocystis*, mainly in the Caribbean and the Andean regions. The sampled areas need to be expanded to establish the transmission rates and genetic characteristics of these microorganisms more accurately. Likewise, it is necessary to note that the results of the present work are an important contribution to explore the frequencies of these parasites in the country but new studies are required to obtain more representative information of the biogeographical regions, increasing the number of samples and including more cities/municipalities to be evaluated in order to establish the real frequency of these microorganisms in the different bioregions. Future studies could consider evaluating samples from different countries in South America as well, which would permit assessment of the frequency of intestinal parasites as well as their STs and assemblages at the continental level. The high frequency of *Blastocystis* and *G. intestinalis* in the samples analyzed likely represents active orofecal transmission involving different hosts in addition to humans, within the life cycles of these protozoa. This might put populations living in vulnerable socioeconomic conditions at risk and it is therefore necessary to implement new strategies for control and prevention of these microorganisms.

## Supplemental Information

10.7717/peerj.8554/supp-1 Supplemental Information 1 Database of the intestinal protozoa. Metadata of the samples collected and analyzed in this study

10.7717/peerj.8554/supp-2 Supplemental Information 2 Raw tpi and gdh Giardia sequences.

10.7717/peerj.8554/supp-3 Supplemental Information 3 Raw 18S sequences of Blastocystis.

10.7717/peerj.8554/supp-4 Supplemental Information 4 Raw 18S sequences of Cryptosporidium.

## Abbreviations

Blast: Basic Local Alignment Search Tool
gdh: glutamate dehydrogenase
Hd: haplotypic diversity
Mega: Molecular evolutionary genetics analysis
S: polymorphic (segregating) sites
SSUrRNA: Small subunit ribosomal ribonucleic acid
ST: subtype
tpi: triose phosphate isomerase

## Additional Information and Declarations

### DNA Deposition

The following information was supplied regarding the deposition of DNA sequences: The raw data is available as a Supplemental File. All sequences are available at GenBank: MN877659–MN877714.

### Data Availability

The following information was supplied regarding data availability: The raw data is available as a Supplemental File.
